# Utility of C3d and C4d immunohistochemical staining in formalin-fixed skin or mucosal biopsy specimens in diagnosis of bullous pemphigoid and mucous membrane pemphigoid

**DOI:** 10.1038/s41598-023-38193-8

**Published:** 2023-07-12

**Authors:** Neha Thakur, Debajyoti Chatterjee, Anubha Dev, Shilpa, Rahul Mahajan, Sanjeev Handa, Dipankar De

**Affiliations:** 1 Department of Dermatology, Venereology and Leprology, Postgraduate Institute of Medical Education and Research, Chandigarh, 160012 India; 2 Department of Histopathology, Postgraduate Institute of Medical Education and Research, Chandigarh, 160012 India

**Keywords:** Diseases, Medical research, Pathogenesis

## Abstract

Bullous pemphigoid (BP) and mucous membrane pemphigoid (MMP) sometimes have overlapping clinical, histopathological, and direct immunofluorescence (DIF) features in the early stages. Complement deposition is an intrinsic component of the patho-mechanism of BP in contrast to MMP. Hence immunohistochemistry (IHC) for C3d and C4d may be helpful in differentiating the two disorders. Seventy-four patients of BP and 18 patients of MMP along with 10 negative controls were enrolled in this study. C3d and C4d IHC was performed in formalin-fixed skin biopsy specimens. C3d IHC staining in BP/MMP had a sensitivity, specificity, positive predictive value (PPV) and negative predictive value (NPV) of 59.2%/41.2%, 100%/100%, 100%/100%, 25.6%/50.0%, respectively. C4d IHC staining in BP/MMP had a sensitivity, specificity, PPV and NPV of 26.8%/17.6%, 100%/100%, 100%/100% and 16.1%/41.7%, respectively. Receiver operator analysis showed utility of C3d in diagnosing both BP [Area under curve (AUC) = 0.8, p = 0.0001] and MMP (AUC = 0.71; p = 0.001). C4d was useful in diagnosis of BP (AUC = 0.5; p = 0.0001), but not MMP (AUC = 0.6; p = 0.064). Hence, C3d is a better diagnostic modality for BP as compared to C4d, whereas C3d and C4d have lower diagnostic importance in MMP. C3d IHC can be employed in diagnosing BP when a second biopsy for direct immunofluorescence (DIF) is not possible or where a facility for IF microscopy does not exist.

## Introduction

The pemphigoid group of disorders are characterized by the presence of autoantibodies against distinct structural components of the dermal–epidermal junction of the skin. Bullous pemphigoid (BP) and mucous membrane pemphigoid (MMP) are two subepidermal blistering disorders with clinical and pathological (both histopathological and immunopathological) overlap. Both these disorders are associated with cellular and humoral autoimmune responses against the proteins in the epithelial basement membrane including hemidesmosomes. In BP, autoantibodies against BP180 and BP230 antigens present in hemidesmosomes interact with various components of the innate immune system including inflammatory cells and complement^1^. Autoantibodies in MMP are formed against BP180, laminin 332, and α_6_β_4_ integrin^2^. Blister formation in MMP is mediated by mechanisms like signal transduction, steric hindrance, and production of reactive oxygen species, similar to the pemphigus group of disorders^1^. This may not be associated with complement activation or deposition.

The differentiation between MMP and BP is made by a combination of clinical, histological, and direct immunofluorescence (DIF) findings. Clinically, both the diseases may resemble each other in the early stages, though MMP patients tend to have predominant mucosal lesions. In the later stages, mucosal scarring may occur which is characteristic of MMP. Histopathology and DIF also show similar features in two. Hence, differentiating MMP from BP may be difficult in the early stages of the disease.

C3d is a stable component of complement degradation that results from the activation of classic and alternative complement pathways. Complement activation plays an important role in the pathogenesis of BP^3^. The mechanisms for blister formation in MMP are independent of complement activation and are direct ones like signal transduction, steric hindrance and production of reactive oxygen species^1^. Hence, immunohistochemistry (IHC) of biopsy specimen from MMP patients may not have complement deposition. Multiple studies have supported the utilization of C3d and C4d immunohistochemistry as an adjunct in the diagnosis of autoimmune bullous disorders^4–10^.

In this study, we aimed to assess the utility of C3d and C4d IHC staining in the diagnosis of BP and MMP and in differentiating between them, when the diagnosis of these two entities were made by a combination of clinical and direct immunofluorescence features as benchmark. This holds importance in resource-poor settings as facility for DIF is not available in majority of places and IHC can be performed where routine histopathology facility is available.

## Materials and methods

**Figure 1:**
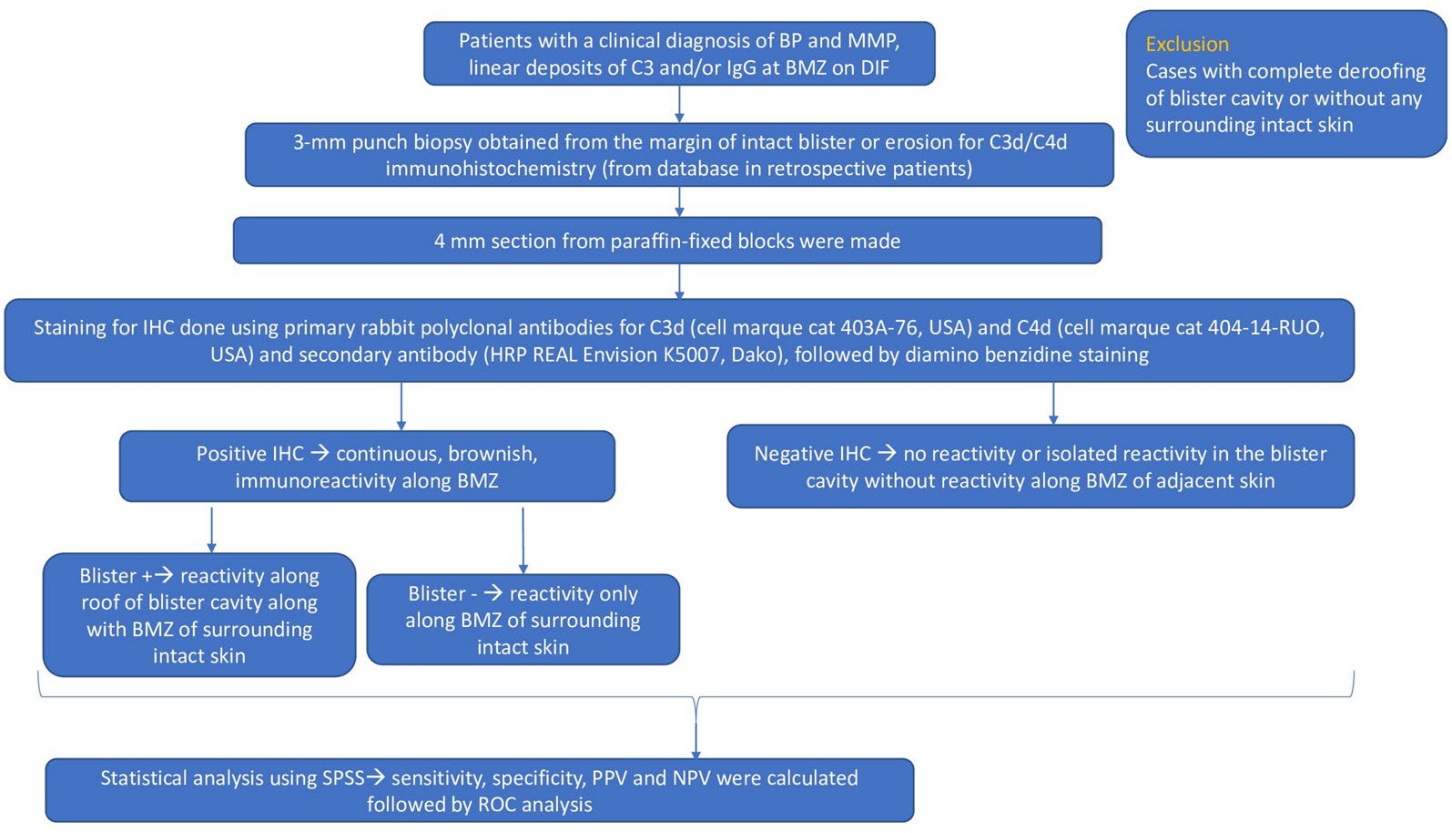
Schematic diagram representing the methodology of the study.

This was a cross sectional study involving patients of BP and MMP registered to the dermatology outpatient department and autoimmune bullous disease clinic of Postgraduate Institute of Medical Education and Research, Chandigarh, India. For patients registered before initiation of the study, archived clinical data and specimens were used. Institute Ethics Committee approvals (Intramural) were obtained before initiation of the study and all research was performed in accordance with relevant guidelines/regulations. Only patients with a clinical diagnosis of either BP or MMP, a DIF of skin/mucosal biopsy positive with linear C3 and/or IgG deposit at the basement membrane zone and availability of paraffin block with adequate tissue for C3d/C4d IHC were included in the study (Fig. 1).

Modification of validated clinical criteria for diagnosis of BP^4^ was used—all of the three clinical characteristics must have been present (absence of atrophic scars, absence of predominant mucosal involvement, and absence of predominant bullous lesions on the neck and head) in presence of linear IgG and/or C3 deposit at the basement membrane zone of the skin on DIF. This modification was required as ‘age at onset > 70 years’ is a criterion in these validated clinical criteria whereas, the average life expectancy in India is 69.4 years^5^.

MMP was diagnosed when there was predominant mucous membrane involvement (oral, ocular, nasal, laryngeal, pharyngeal, genital and peri-anal) with intact blister or erosion with or without scarring along with linear IgG and/or C3 deposit at the basement membrane zone of the mucosa on DIF.

### Direct immunofluorescence

A 3-mm punch biopsy was obtained under local anesthesia from the margin of an intact blister or erosion for routine histopathology and C3d/C4d immunohistochemistry. Another 3-mm punch biopsy was taken from peri-lesional area for direct immunofluorescence. Deposition of different immune-reactants was reported as linear positivity along dermo-epidermal junction in a semi- quantitative manner.

### C3d and C4D immunohistochemistry

C3d and C4d IHC was done for patients of BP and MMP, using standard protocol. Primary antibodies used were C3d (Cell marque cat 403A-76, USA) and C4d (Cell marque cat 404-14-RUO, USA) rabbit polyclonal antibody. Positivity of IHC was indicated by continuous, brownish, immunoreactivity along basement membrane, including positivity along the roof of the blister cavity (will be further referred to as Blister+). The intensity of deposition was graded as 1+, 2+ or 3+. Cases where there was complete de-roofing of the blister without any normal adjacent skin; IHC could not be interpreted using the algorithm described above and such cases were excluded from analysis. It was interpreted as negative if there was no reactivity. Since the blister cavity contains fibrin deposition, it can give false positive staining. Hence isolated positivity in the blister cavity without reactivity along dermo-epidermal junction in the adjacent skin was also considered negative. Additionally, immunoreactivity along the basement membrane of intact surrounding skin and not along the blister roof or floor, were analyzed separately and will be further referred to as “Blister−”.

### Negative control for immunohistochemistry

Ten overlying skin sections of total mastectomy specimens with axillary clearance for carcinoma breast, without cutaneous involvement by tumor or significant inflammation were used as negative controls.

### Statistical analysis

The data was analysed using Statistical Package for Social Sciences (SPSS for Windows, version 28, IBM, New York, USA). Normality of the quantitative data was assessed by Kolmogorov Smirnov test. The descriptive data was presented as mean ± standard deviation (SD) or median/inter quartile range, depending upon the normality of the data. Qualitative or categorical data was described as frequencies and proportions. The Chi-square test or Fischer’s exact test was used for comparison between groups with categorical data. The sensitivity, specificity, positive predictive value, and negative predictive value was calculated with standard formulas. The p-value of less than 0.05 was considered as statistically significant. The performance of IHC was compared against combined clinical and DIF diagnosis for both BP and MMP. Receiver operator curve (ROC) analysis was performed for C3d and C4d in both BP and MMP and AUC was calculated to compare their diagnostic significance. Youden index was measured as per the formula “sensitivity + specificity − 1”. Diagnostic accuracy was calculated as per the formula “(true positive + true negative)/all subjects”.

### Study approval

This study protocol was reviewed and approved by intramural institutional ethics committee of PGIMER, Chandigarh (approval number - INT/IEC/2021/SPL-238).

### Consent to participate

Written informed consent was obtained from participants who were included prospectively.

## Results

A total of 92 patients were enrolled in the study. Of them, 74 (80.4%) patients had a diagnosis of BP and 18 (19.6%) of MMP.

The median (IQR) age was 64.5 years (52.75–72.00). Forty-three patients (46.7%) were male and 49 (53.3%) were female. Among patients of BP, 36 were male and 38 were female and among MMP, 7 were male and 11 were female.

Histopathology findings have been summarized in Supplementary (Suppl.) Table 1. DIF results have been summarized in Supplementary Table 2. IgA positivity was much more frequent in MMP in comparison to BP and this difference almost reached statistical significance (p value = 0.05).

### Immunohistochemistry

**Figure 2:**
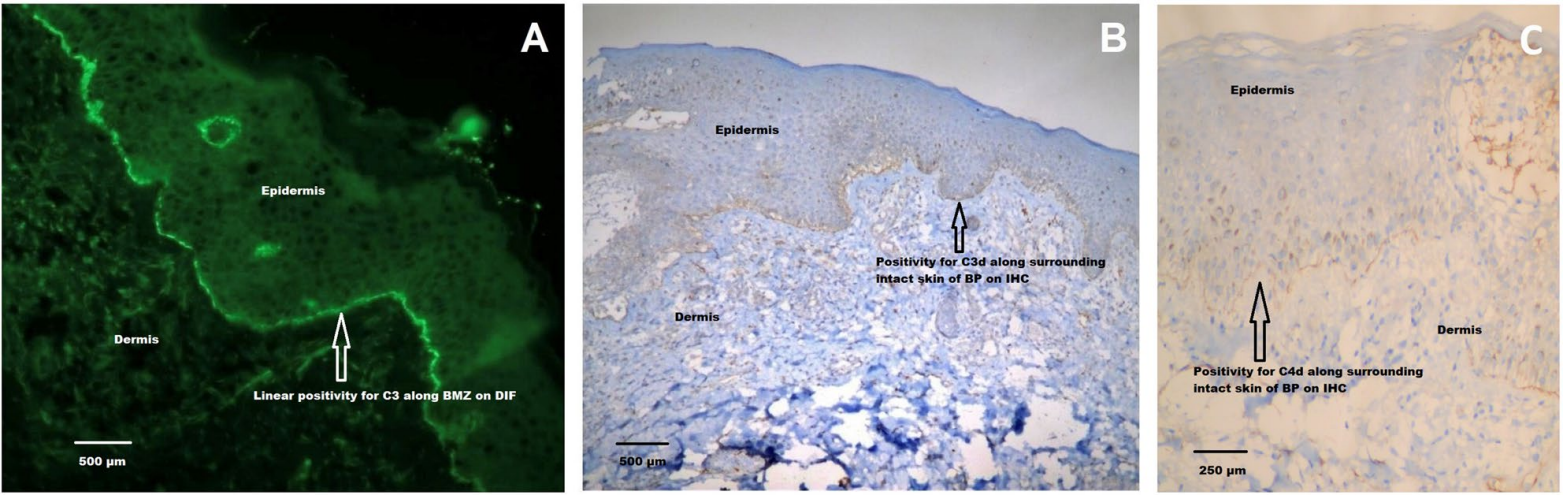
(**a**) Direct immune-fluorescence of a skin biopsy specimen in a patient of bullous pemphigoid depicting linear deposits of C3 at the basement membrane zone (×10) (**b**) C3d immunohistochemistry performed on the skin biopsy specimen of the same patient depicts immunoreactivity along the basement membrane zone in the surrounding intact skin (×10) (**c**) C4d immunohistochemistry performed on the skin biopsy specimen depicts immunoreactivity in the basement membrane zone in the surrounding intact skin (×20).

Results of IHC for C3d and C4d have been summarized in Table 1 and representative image of C3d and C4d positivity has been depicted in Fig. 2. On Blister+ analysis, C3d IHC was positive in 59.2% patients of BP and 41.2% patients of MMP. C4d IHC was positive in 26.8% patients of BP and 17.6% patients of MMP. However, on Blister− analysis, IHC for C3d was positive in 44.3% patients of BP and 27.7% patients of MMP. For C4d, IHC was positive in 15.1% patients of BP and negative in all patients of MMP. The frequency of C3d positivity on IHC was significantly higher in BP as compared to MMP on Blister− analysis (p value = 0.043).

**Table 1:** Immunohistochemistry in patients of BP and MMP.

IHC	BP [n (%)]	MMP [n (%)]	P value
Blister+			
C3d	n = 71*	n = 17*	
Positive	42 (59.2%)	7 (41.2%)	0.18^a^
Negative	29 (40.8%)	10 (58. 8%)	
C4d	n = 71	n = 17	
Positive	19 (26.8%)	3 (17.6%)	0.44^a^
Negative	52 (73.2%)	14 (82.4%)	
Blister−			
C3d	n = 70#	n = 17	
Positive	32 (45.7%)	3 (27.7%)	**0.043**^a^
Negative	38 (54.3%)	14 (82.3%)	
C4d	n = 70	n = 17	
Positive	11 (15.7%)	0 (0%)	0.113^b^
Negative	59 (84.3%)	17 (100%)	

Significance values are in bold.

^a^Chi square test.

^b^Fischer’s exact test.

*3 biopsies in BP group and 1 in MMP group could not be interpreted due to inadequate tissue.

^#^4 biopsies in BP group could not be interpreted due to inadequate tissue. One biopsy which was deemed adequate in Blister+ analysis was considered inadequate in Blister− analysis due to lack of surrounding non-separated skin.

Amongst the patients with positive DIF for C3, C3d IHC was positive in 58.6% and C4d IHC in 27.1% (Blister+ analysis). On Blister−, C3d IHC was positive in 39.4% and C4d IHC in 15.5%. None of the participants who had negative C3 DIF had positive C4d IHC but 42.8% patients (including both BP and MMP) were positive for C3d IHC on Blister+ analysis and 23.8% were positive in Blister− analysis.

### Sensitivity, specificity, positive predictive value and negative predictive value of C3d and C4d in diagnosis of BP and MMP against negative control

The parameters measuring the diagnostic accuracy of IHC have been described in Table 2. On Blister+ analysis, the sensitivity of C3d was 59.2% in BP and 41.2% in MMP and that of C4d was 26.8% in BP and 17.6% in MMP. Specificity and positive predictive value were 100% across for both C3d and C4d in BP as well as MMP. The overall diagnostic accuracy of C3d in BP was 64.2% and was 62.9% in MMP and the accuracy was lower for C4d (35.8% and 48.1% in BP and MMP respectively).

**Table 2:** Diagnostic utility of C3d and C4d IHC in the diagnosis of BP and MMP.

Variable		Sensitivity	Specificity	PPV	NPV	Youden index	Diagnostic accuracy	Area under receiver-operator curve	P value
Blister+									
IHC: C3d	BP	59.2% (46.8–70.7)	100.0% (69.2–100.0)	100.0%	25.6% (20.7–31.3)	0.6	64.2%	0.8	**0.0001**
	MMP	41.2% (18.4–67.1)	100.0% (69.2–100.0)	100.0%	50.0% (40.2–59.8)	0.4	62.9%	0.71	**0.001**
IHC: C4d	BP	26.8% (16.9–38.6)	100.0% (69.2–100.0)	100.0%	16.1% (14.3–18.1)	0.3	35.8%	0.5	**0.0001**
	MMP	17.6% (3.8–43.4)	100.0% (69.2–100.0)	100.0%	41.7% (36.4–47.1)	0.2	48.1%	0.6	0.064
Blister−									
IHC: C3d	BP	44.3% (32–57)	100.0% (69–100)	100.0%	20.4% (10–34)	0.44	51.2%	–	–
	MMP	17.6% (4–43)	100.0% (69–100)	100.0%	41.7% (22–63)	0.18	48.1%		
IHC: C4d	BP	15.1% (8–25)	100.0% (69–100)	100.0%	13.9% (7–24)	0.15	25.3%	–	–
	MMP	0.0% (0–19)	100.0% (69–100)	NA	35.7% (19–56)	0	35.7%	–	–

Significance values are in bold.

Youden index = sensitivity + specificity − 1.

Diagnostic accuracy = (true positives + true negatives)/all subjects.

On Blister− analysis, IHC for C3d showed a reduced sensitivity of 44.3% in BP and 17.6% in MMP with a reduced diagnostic accuracy of 51.2% and 48.1% in BP and MMP, respectively. The sensitivity of C4d in BP was even lower (15.1%), with a diagnostic accuracy of 25.3% and had minimal role in diagnosing MMP (sensitivity 0).

### Receiver operator curve (ROC) analysis

**Figure 3:**
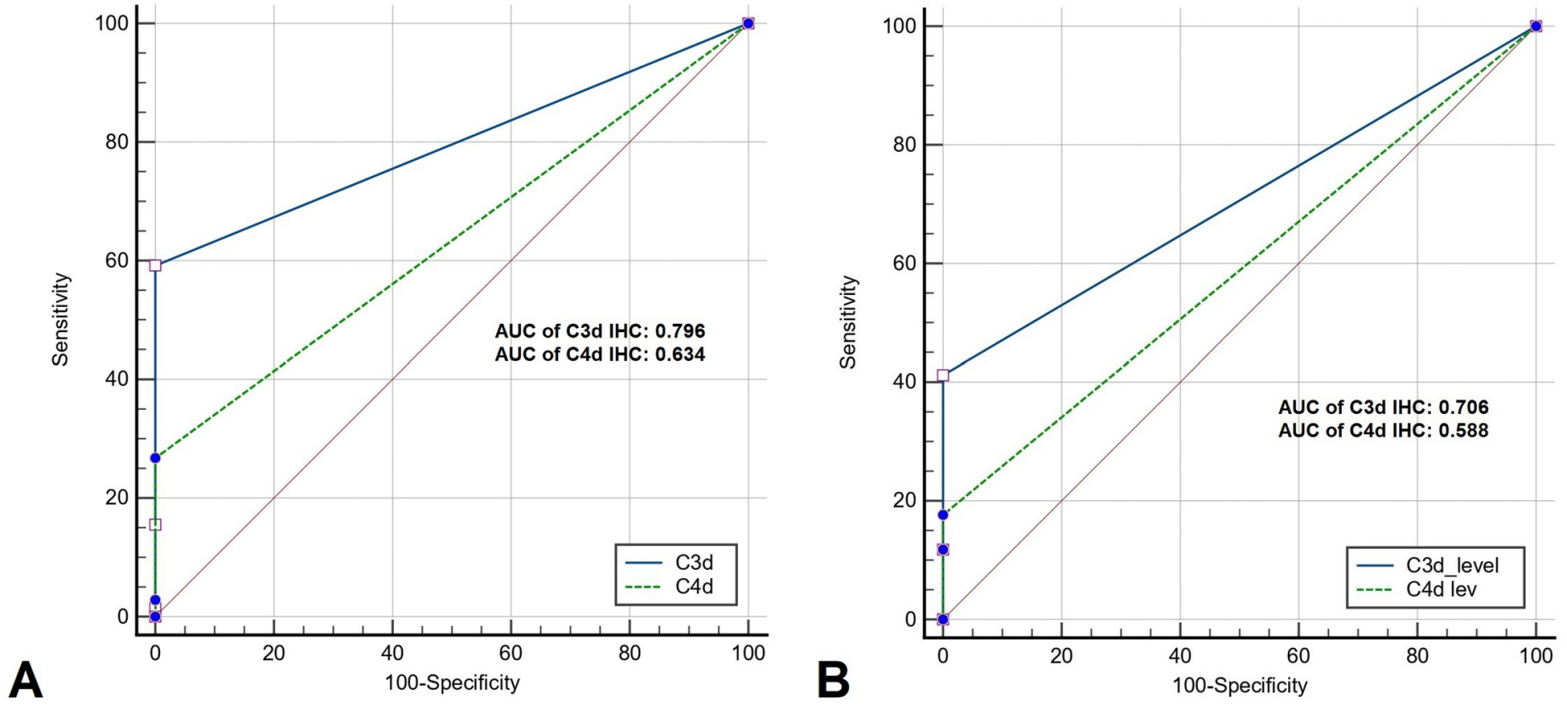
(**a**) Comparison of ROC curves of C3d and C4d in BP (**b**) Comparison of ROC curves of C3d and C4d in MMP.

The area under curve (AUC) achieved statistical significance for the utility of C3d in diagnosing both BP (p = 0.0001) and MMP (p = 0.001). While AUC of C4d in diagnosis of BP was also significant (p = 0.0001), it was not significant in the diagnosis of MMP (p = 0.064). On comparative analysis of ROC of C3d and C4d in BP (Fig. 3A), the difference in AUC was 0.162 (0.09–0.23) [p = 0.0001]. On a similar analysis in MMP (Fig. 3B), the AUC difference was 0.12 (− 0.02 to 0.25) [p = 0.08].

## Discussion

Pemphigoid diseases are a group of sub-epidermal blistering disorders of skin and/ or mucosa in which autoantibodies are formed against the structural building blocks of the dermal–epidermal junction (DEJ) of the skin and/ or mucosa. Jordon and colleagues were the first to describe autoantibodies in serum and in skin among patients of pemphigoid^6^. Since then, the identification of specific autoantibodies bound to epithelium and skin has become the standard for diagnosis of pemphigoid disorders.

Both BP and MMP are diagnosed based on clinical, histopathological and DIF findings. MMP tends to have more mucosal lesions, including the oral, conjunctival, ano-genital and upper aero-digestive tract mucosa. Oral lesions are seen in 10–20% of cases of BP. Skin lesions heal with hypopigmentation and rarely with scarring and milia^7^. The lesions of MMP may heal with residual scarring at the later stages leading to symblepharon, trichiasis, neovascularization, blindness, laryngeal stenosis, etc.^2^. Early lesions of MMP on histopathology may have a sub-epidermal cleft with lympho-histiocytic infiltrates with granulocytes and plasma cells, while the older lesions have fibroblast infiltrates and fibrosis. In BP, the infiltrate may be eosinophil rich. Other modalities like DIF (linear deposition of IgG and or C3 at DEJ), IIF and ELISA (detecting autoantibodies against BP 180 and 230) support the diagnosis of both BP and MMP. Deposition of IgA is more frequent in MMP than BP.

Differentiating BP and MMP in the early stages of the disease is often difficult, based on clinical features, skin/mucosal biopsy histopathology, DIF, IIF and also by ELISA, as the features can be similar. At the later stages, they can be differentiated clinically, based on predominant mucosal involvement and possible scarring in MMP. However, the disfiguring complications warrant the early differentiation of BP from MMP for timely intervention and prevention of scarring.

Conventionally, DIF studies are used to demonstrate the deposition of immunoglobulin and complement in biopsy specimens^8^. IHC is not widely used for the diagnosis of autoimmune bullous diseases. However, this approach has additional advantages, particularly for those cases where frozen sections are not available for DIF and second biopsy cannot be taken^9^. Facility for DIF is not available in many places/ laboratories. Analyzing IHC with a standard light microscope helps in visualization of cellular morphology, overcoming the shortcomings of fluorescent imaging. While Velez et al.^10^ reported around 98% correlation between DIF and IHC as a diagnostic modality in patients of multiple autoimmune bullous disorders (pemphigus vulgaris, pemphigus foliaceous, BP and dermatitis herpetiformis), other studies like Glauser et al.^9^ reported much lower sensitivity and specificity of IHC as compared to DIF in immunobullous dermatoses. Oh et al. reported a similar sensitivity of IHC and DIF in the diagnosis of BP (80.8% vs 84.3%) but a higher specificity of IHC over DIF (83.3% vs 75%)^11^. They also demonstrated the utility of IHC in diagnosing cases of DIF negative BP. Similarly, Wang et al. demonstrated similar sensitivity, specificity, PPV and NPV of C3d IHC as compared to DIF and IIF^12^. In order to further explore its utility, we used IHC to detect complement deposition and have compared its sensitivity, specificity, PPV and NPV with respect to DIF as gold standard for this comparison. Amongst the patients whose DIF was positive for C3, C3d IHC was positive in 58.6% and C4d IHC in 27.1% patients. The sensitivity and specificity of IHC for C3d and C4d was lower than DIF for C3 in both BP and MMP biopsies, which are more in keeping with the findings of Glauser et al. Interestingly, 27.3% patients who were negative for C3 on DIF were positive for C3d on IHC (Blister+).

The blister formation in BP is predominantly mediated by constant region of IgG1 and IgG4^13^. IgG1 promotes the deposition and activation of complements, whereas IgG4 results in activation of leucocyte and dermal–epidermal separation. The studies which have described the diagnostic utility of IHC for C3d and C4d deposition in autoimmune bullous diseases are summarized in Supplementary Table 3. The differences of sensitivity of complement IHC in AIBDs among different studies could be because of different clones of antibodies used and the method of interpretation.

In MMP, the blister formation is directly mediated by the variable region of antibody, including signal transduction and steric hindrance^2^. The binding of autoantibodies (predominantly IgG) against the antigens (BP 180, integrin or laminin 332) leads to disruption of interaction of these molecules with other DEJ components. Hence complement deposition is likely to be less frequent in biopsies of MMP. The findings of our study reflect the same where C3d by IHC was less commonly positive in MMP in comparison to BP and C4d was found to be even less frequently positive in patients of MMP as compared to BP. A study by Shimanovich et al.^14^ in patients of MMP to determine the diagnostic utility of either C3d or C4d IHC had a sensitivity of around 50%. The findings of our study (Blister+) were consistent with that of Shimanovich et al.; C3d IHC had a sensitivity of 41.2% for MMP, but the sensitivity of C4d IHC was much lower.

The sensitivity of C3d and C4d IHC in the diagnosis of BP and MMP was observed to be higher in the Blister+ analysis as compared to Blister− and so was the negative predictive value. Hence it might be worthwhile to consider immunoreactivity along the blister roof and floor along with that of the basement membrane zone of the surrounding non-separated skin for a greater diagnostic yield.

Comparing the usefulness of C3d and C4d IHC in BP and MMP, both C3d and C4d have a higher sensitivity as well as negative predictive value in diagnosing BP as compared to MMP. The frequency of C3d was significantly higher in BP as compared to MMP on Blister− analysis. This could possibly indicate the utility of C3d as a marker to differentiate between BP and MMP (sensitivity of 44.3% vs 17.6% respectively), when immunoreactivity only in the surrounding intact skin is considered. Overall, C3d is significantly more useful than C4d in diagnosis of BP. Even though the sensitivity and AUC of C3d is higher than C4d in MMP, this difference did not achieve statistical significance.

## Conclusion

IHC for C3d and C4d has been explored as modalities to aid in the diagnosis of BP and MMP. It has certain advantages over DIF that makes it a useful diagnostic tool. The sensitivity for C3d IHC for BP was lower than the previous studies but the specificity was similar. C3d is a better diagnostic modality for BP, whereas C3d and C4d have lower diagnostic importance in MMP. Consideration of positivity of C3d and C4d on deposition on roof or floor of blister alone increases the sensitivity and subsequently the diagnostic importance of C3d and C4d on IHC. C3d IHC can be employed in diagnosing BP when a second biopsy for direct immunofluorescence (DIF) is not possible or where a facility for IF microscopy does not exist.

## Supplementary Information

Supplementary Tables.

## Data Availability

Data will made available upon reasonable request from the authors.
