# Supplementary material for: Utility of C3d and C4d immunohistochemical staining in formalin-fixed skin or mucosal biopsy specimens in diagnosis of bullous pemphigoid and mucous membrane pemphigoid
Source: Sci Rep. 2023 Jul 12;13:11283. doi: 10.1038/s41598-023-38193-8 (PMC10338454; doi:10.1038/s41598-023-38193-8)
Supplement: Supplementary file 1 — Supplementary Tables. [file 41598_2023_38193_MOESM1_ESM.docx]

**Suppl. Table 1: Histopathological features of biopsy specimens in studied patients**

| **Parameters** | **BP [n (%)]** | **MMP**  **[n (%)]** | **P value** |
| --- | --- | --- | --- |
| **Subepidermal blister (n=90) *** | n=73 | n=17 |  |
| Split present | 64 (87.7%) | 12 (70.6%) | 0.08^1^ |
| No split | 9 (12.3%) | 5 (30.4%) |  |
| **Eosinophils (n=90) *** | n=73 | n=17 |  |
| 0 | 38 (52.0%) | 10 (58.8%) | 0.43^1^ |
| 1+ | 18 (24.7%) | 6 (35.3%) |  |
| 2+ | 15 (20.5%) | 1 (5.9%) |  |
| 3+ | 2 (2.7%) | 0 (0.0%) |  |
| **Scarring/Fibrosis (n=91)** | n=74 | n=17 |  |
| Present | 6 (8.1%) | 2 (11.7%) | 0.64^2^ |
| Absent | 68 (91.9%) | 15 (88.3%) |  |

^1^ Chi square test

^2^ Fischer’s exact test

###### * Two biopsies (one in BP and one in MMP group) could not be interpreted due to lack of epidermis in the biopsy specimen

###### Suppl. Table 2 : Direct Immunofluorescence in patients of BP and MMP

| **DIF** |  | **BP [n (%)]**  **n=74** | **MMP [n (%)]**  **n=18** | **P value** |
| --- | --- | --- | --- | --- |
| **C3** | **Positive** | 57 (77%) | 14 (77.8%) | 1.000^1^ |
|  | **Negative** | 17 (23%) | 4 (22.2%) |  |
| **IgG** | **Positive** | 65 (87.8%) | 17 (94.4%) | 0.680^1^ |
|  | **Negative** | 9 (22.2%) | 1 (5.6%) |  |
| **IgM** | **Positive** | 5 (6.7%) | 1 (5.6%) | 1.000^1^ |
|  | **Negative** | 69 (93.3%) | 17 (94.4%) |  |
| **IgA** | **Positive** | 12 (16.2%) | 7 (38.9%) | 0.05^2^ |
|  | **Negative** | 62 (83.8%) | 11 (61.1%) |  |

^1^Fischer’s exact test

^2^Chi square test

**Suppl. Table 3: Summary of the studies describing the utility of IHC and IHC for C3d and C4d in diagnosis of BP and MMP**

| **Authors of study** | **Disorders studied** | **Sample size** | **Study design** | **Results** | **Conclusion** |
| --- | --- | --- | --- | --- | --- |
| Velez et al | BP, PV, PF, DH | BP-34  PV-4  PF-38  DH-14 | Retrospective study;  IgG, IgM, IgA, IgD, IgE, Kappa light chains, Lambda light chains, C3c, C1q, C3d, albumin and fibrinogen by IHC | Diagnostic correlation between IHC and DIF-IIF was 98% | IHC is as reliable as DIF or IIF for the diagnosis of AIBD |
| Magro et al | BP, PV, other collagen vascular diseases and vasculopathies | BP-17  PV-11  Collagen vascular diseases and vasculopathies-129  Controls- 23 | Both prospective and retrospective;  C3d and C4d IHC was done | BP- homogenous DEJ C3d in all patients but C4d  was negative; 100% concordance with DIF  PV- intercellular C3d and C4d (similar to pattern on DIF) (9/11) | C3d and C4d assay significant in the assessment of AIBD |
| Glauser et al | BP | 38 (51 biopsies) | Retrospective study; IHC was compared to DIF | By IHC, deposits of C3d, IgG, IgM, IgE and IgA were found in significantly lesser percentage of biopsies as compared to DIF | IHC not sufficiently sensitive to replace DIF studies for diagnosis of BP; |
| Pfaltz et al | Various AIBD (BP, PV, PF, PG  LAD  DH, mixed, IgA pemphigus) | AIBD-65  Controls-44 | Retrospective study; DIF and C3d IHC was performed | C3d has a 97% sensitivity, 100% PPV, 98%  NPV in diagnosis of BP | C3d IHC is a  helpful adjunct in the diagnosis of BP,  negative result makes the diagnosis of a BP unlikely |
| Wang et al | BP | BP-27  Other disorders-24 | Retrospective study, compared C3d IHC with DIF and IIF, using anti-BP180 or anti-BP 230 ELISA results as the gold standard | C3d IHC, DIF and IIF had similar sensitivity specificity, PPV and NPV for BP | C3d IHC on fixed tissue provides similar diagnostic utility to immunofluorescence and ELISA |
| Oh et al | BP and other AIBD | Clinically and DIF confirmed BP=43  DIF negative BP=9  Other AIBD= 36 | Retrospective study, diagnostic utility of C3d, C4d and IgG IHC in BP | Positive C3d, C4d, or IgG IHC in 86% DIF-confirmed BP cases; sensitivity of IHC similar to DIF (80.8% vs. 84.3%), but specificity was higher (83.3%  vs. 75.0%);  5/9 DIF negative BP had positive IHC | IHC may be a useful alternative to diagnosis of BP. |
| Chandler et al | BP vs EBA | 10 | Retrospective study;  IHC for polyclonal C4d | Linear basement membrane  deposition of C4d in formalin-fixed paraffin-embedded tissue in 7/9 cases diagnosed as BP vs. EBA by DIF | Formalin-fixed paraffin embedded  tissue can be stained for the C4d to show  deposition |
| Shimanovich et al | MMP | 34 (50 biopsies) | Retrospective study; IHC for C3d, C4d, IgG and IgA was done | Sensitivity of C3d or C4d IHC was 50%, sensitivity for mucosal biopsy was lower | C3d or C4d IHC is a helpful screening procedure for cases of suspected MMP where frozen tissue is not readily available. |

#### PV-Pemphigus vulgaris; PF-Pemphigus foliaceous; DH-Dermatitis herpetiformis; AIBD- Autoimmune bullous dermatoses; PG- Pemphigoid gestationis; LAD- Linear IgA disease; EBA- Epidermolysis bullosa aquisita
